# Girls, mental health problems, and offending: findings from a community sample

**DOI:** 10.1186/s13034-025-00907-3

**Published:** 2025-05-19

**Authors:** Linn Persson, Anna-Karin Ivert

**Affiliations:** Department of Criminology, Malmö University, 205 06 Malmö, Skåne Sweden

**Keywords:** Girls, Mental health problems, Offending, Community sample, Deviant peers, Parents

## Abstract

**Background:**

Mental health problems (MHPs) are associated with youth offending, but research on MHPs among specifically offending girls, particularly in community settings, is limited.

**Aims:**

To explore if MHPs were more common among adolescent girls who reported committing crimes compared to those who did not, as well as to investigate how different MHPs were associated with offending, and examine the potential effects of parental relationships, parental monitoring, and association with deviant peers.

**Methods:**

Data were drawn from the Malmö Individual and Neighbourhood Development Study (MINDS), a longitudinal study which comprises a random sample of 525 adolescents (~ 20%) born in 1995 and living in Malmö, Sweden, in 2007. The current study included the 240 girls that participated in wave two (age 16) and three (age 17) of data collection. Data were collected using a self-reported questionnaire. Independent samples T-tests analysed differences in MHPs between offending and non-offending girls. Pearson’s correlation test and logistic regressions examined the association between MHPs and offending and how these associations were affected by parental relationship, parental monitoring, and deviant peers.

**Results:**

Offending girls had higher levels of MHPs than non-offending girls, with the most significant differences in hyperactivity and externalising problems. Logistic regressions partly confirmed these findings, showing strong associations between externalising problems and offending. Internalising problems showed mixed results in their association with offending.

**Conclusion:**

Girls who had offended had higher levels of both internalising and externalising MHPs compared to those who had not offended. This indicates that measures to prevent youth crime should acknowledge MHPs. Overall, more research is needed on girls' MHPs and offending, particularly on the association between internalising problems and offending.

**Supplementary Information:**

The online version contains supplementary material available at 10.1186/s13034-025-00907-3.

## Introduction

In criminology, it has historically been uncommon for studies to focus only on girls’ offending, and therefore, there are still unanswered questions about why girls offend and if there are risk factors that are specific for them [35]. However, during the past decades, there has been an increase in girls who offend (e.g., [36, 57, 61]) and an increased number of girls in the juvenile justice system [17, 23, 80]. This development can also be seen in Sweden, with just over 50% of ninth-grade school girls in Sweden reporting that they have committed some offence during the past year in the National School Survey on Crime 2023 [75]. Previous studies on mixed gender samples have provided a general basis for understanding girls’ offending, and the studies have found that, for example, mental health problems (MHPs) seem to be an important variable for explaining offending (e.g., [1, 10, 13, 44]). For example, having externalising problems [15] and problems with several different sorts of MHPs (Siponen et al., [67]) has been found to increase the risk of offending, and among youth that commit a high number of crimes and continue to offend in adulthood the rate of MHPs is generally higher than among those who do not (e.g., [49, 51]). The more limited amount of research focusing specifically on girls has shown similar results as the research on mixed samples (e.g., [51]), but also notable differences [39]. For example, besides externalising problems, internalising problems have also been found to be common among offending girls [73]. At the same time, a growing number of girls report having problems with some types of MHPs (e.g., [74, 85]), especially when it comes to internalising problems [12]. The fact that girls are experiencing an increase of both MHPs and offending calls for more knowledge about the associations between these factors. Knowing more could provide better explanatory models for why some girls offend and foster better preconditions for preventive measures, which in turn can contribute to better living conditions for girls. Previous research has indicated that variables such as parent–child relationship, parental monitoring, and peers might be of high importance for youth MHPs (e.g., [9, 38, 70]), as well as offending (e.g., [24–26]). Since they might affect an offending outcome, they need to be controlled for. Therefore, in the current study, we explore if some MHPs are more common among girl offenders, how different MHPs are associated with offending, and if these associations are affected by parent–child relationship, parental monitoring, and peers.

## Background

Initially, this will provide a description of how MHPs are defined in the current study, which is followed by a presentation of findings from previous research on the association between offending and MHPs. Lastly, it presents findings from previous research on the risk factors of both crime and MHPs that are examined in the current study, the parent–child relationship, parental monitoring, and associations with peers.

### Study definition of mental health problems

In this study, the concept of MHPs is based on the definitions from the WHO [86] and the American Psychiatric Association [5], including disorders, disabilities, and impairments originating from deviations in brain functioning, as well as the consequences and problems of these disorders and impairments that, among other things, can affect behaviour, emotions, and well-being. In the current study, we explore a range of self-reported symptoms, such as symptoms of hyperactivity or emotional problems; and symptoms that can also be divided into the broader symptom subgroups of internalising and externalising problems. These symptoms possibly indicate a mental disorder according to the current diagnostic symptoms, but not necessarily. Consequently, we use the broader term MHP rather than, e.g., mental disorder. This approach allows for a broader understanding of which types of problems and symptoms are most relevant for girls' offending, while also potentially reducing stigma related to diagnoses.

### Mental health problems and offending

#### Findings from studies with mixed gender samples

Much of the previous research providing knowledge about the association between girls’ MHPs and offending has been conducted on samples with both girls and boys, and the amount of research focusing on only girls is still quite low. Many of the mixed sample studies have been conducted on clinical samples (e.g. [48]) or within the juvenile justice system, where it has been found that between 52 and 70% of youth suffer from some form of MHP (e.g., [11, 46, 79]), with especially externalising problems being common [13]. Even though there are fewer studies conducted on community samples, the existing ones show similar results as the ones from the juvenile justice system, indicating that externalising problems are important for understanding youth offending. For example, Moffitt [49, 50] and Moffitt et al. [51] found that individuals who followed a trajectory of persistent and frequent crime involvement (life course persistent offenders) often had externalising problems like neurodevelopmental problems (which are problems with the development of the nervous system in both the brain and spinal cord, potentially causing, for example, hyperactivity, inattention, and emotional dysregulation) and conduct problems. Moffitt’s [49, 50] and Moffitt’s et al. [51] findings have been supported in more recent community-based studies, which have found that externalising problems are more common than internalising problems among youth offenders [42], that neurodevelopmental problems are associated with offending among youth at ages 9–12 [62], and that youth having unmedicated ADHD (and thus unmedicated problems with hyperactivity, concentration, and impulsivity) had a higher risk of offending [52]. However, even though there already are indications of associations between externalising problems and youth offending, there are also studies indicating that the relationships between youth MHPs and offending are quite complex and need to be further studied (e.g., [47, 67]). For example, Anderson et al. [6] found that depression may increase the risk of property crimes, but not violent crimes, and a recent Swedish national population-based register study by Siponen et al. [67] found that comorbidity between several problems and diagnoses, including both internalising and externalising problems, may increase the risk of criminal conviction.

### Findings from studies focusing on girls

#### Findings from justice settings or clinical populations

Even though studies on mixed gender samples have contributed with important knowledge about the associations between girls’ MHPs and offending, the few studies that have focused on and examined only girls on their own might be of even greater importance for understanding the associations between girls’ MHPs and offending. As with studies including both genders, it is most common that studies focusing on girls have been conducted in the juvenile justice setting or on clinical samples. To start, and notably, it has been suggested that MHPs might be more important to explain girls’ offending rather than boys’, as it was found that MHPs may lead to a higher risk of being convicted of offences among girls than boys in a longitudinal, registry-based sample from Sweden [67]. Similar indications have been found among adult females in the justice setting, showing that no matter their ethnic background and age, female offenders have higher rates of MHPs compared to male offenders (e.g., [14, 28, 31]). Moreover, up to 80% of women in prison have some form of MHPs [87], and they are up to five times more likely to suffer from MHPs than women in the general population [78]. Even though these findings refer to adult females, similar results have been found among girls in the juvenile justice system, with as much as 74% of girls suffering from one or more MHPs [73, 80]. Further, like the findings from mixed samples, findings from studies on only girls in the juvenile justice system also indicate that externalising problems could be important among offending girls where, for example, it has been found that 46% of detained girls had disruptive behaviour problems [73], that girls were both more likely to be diagnosed with oppositional-defiant disorder (ODD) or conduct disorder (CD) [19], and with being more violent towards staff (thus showing tendencies of disruptive behaviour) than boys [76].

Previous research from the juvenile justice setting with its focus on only girls has, however, also shown that not only externalising problems are associated with offending (e.g., [73]), but importantly and interestingly that also internalising problems might be important for fully understanding girls’ offending [39]. For example, in a longitudinal study where detained girls were assessed two times during a 4.5 year time period, Van der Molen et al. [80] identified three trajectories of disruptive behaviour, with those in the high-risk group facing increased risks of not only aggression, but also depression, self-harm, and PTSD. Moreover, among girls in the juvenile justice system, it has been found that 19% of the girls had depression [73], 47% to 72% of the girls had anxiety disorders [14, 56], and Trulson et al. [76] reported that girl offenders were likely to have experienced risk factors for internalising disorders such as sexual, physical, and emotional abuse. However, there is research indicating that a prison environment can affect female mental health, especially depression and anxiety, negatively (e.g., [34, 77]), which might possibly contribute to the high levels of these problems in juvenile justice settings, making it difficult to fully understand how internalising problems are associated with offending among girls.

Notably, many of the studies conducted in justice or clinical settings rely on cross-sectional designs, which affect the possibility to determine whether MHPs precede offending behaviour or are a consequence of it—or perhaps are influenced by shared underlying risk factors. This is something that needs to be considered when drawing conclusions in relation to the causal order of MHPs and criminality.

#### Findings from community-based studies

Overall, community-based studies show similar results as studies conducted in the juvenile justice setting; MHPs can also be a risk factor of girls’ offending in community samples. However, compared to studies conducted in justice or clinical settings, there are more studies with longitudinal designs. For instance, a school-based study tracking girls from middle and high school into adulthood found that severe MHPs, such as anxiety and depression, were significant predictors of offending [64]. Additionally, a longitudinal study following serious adolescent offenders through their transition to adulthood revealed that girls with MHPs (also, for example, depression and anxiety) and trauma symptoms were more likely to engage in persistent criminal behaviour. Moreover, the study highlighted that MHPs, combined with substance abuse and family dynamics, were key factors influencing continued offending among girls [54]. Farrington [24] also conducted a longitudinal study following both girls and boys from childhood into adulthood, focusing on the development of offending behaviour. When examining only the included girls, Farrrington [24] found that girls with early signs of emotional dysregulation and MHPs, such as anxiety and depression, were more likely to engage in delinquent behaviour and MHPs were also found to interact with other risk factors, such as family instability and exposure to violence, to increase the likelihood of criminal activity [24]. Similar to Farrington [24], Moffitt [51] examined only the girls’ offending trajectories in more detail and separately from boys, and found that girls who start offending early exhibit the same externalising problems and follow the same trajectory paths as found in the mixed gender studies presented earlier [7, 8, 51, 68]. Moreover, regarding girls’ trajectories, Andersson et al. [8] interestingly identified a small group of females with adult-onset offending that has not been seen to the same extent in mixed sample studies, indicating important gender-specific characteristics of girls’ offending development and trajectories. Also, in the Girls Group Study, conducted by the U.S. Department of Justice, developmental pathways to delinquency in girls were examined. This study identified that girls with MHPs and trauma had higher rates of offending and recidivism compared to those without [69].

#### The complexity of different MHPs for girls’ offending

However, even though there are indications from previous findings that MHPs seem to be an important risk factor of girls’ offending, the associations between different MHPs and offending might need to be problematised further due to potential complex interactions between both MHPs and offending. For example, in regard to ADHD and its association with offending (e.g., [52]), explanations of the association between ADHD and offending often focus on externalising problems such as hyperactivity and impulsivity [2], but there are suggestions that also internalising problems must be considered among offending girls with the diagnosis (e.g., [41, 47]). Girls with ADHD have been found to report less externalising behaviour compared to boys, and more depressive problems [41], and it has been suggested that ADHD is underdiagnosed among girls, with one explanation being that ADHD symptoms are misinterpreted or overshadowed by other MHPs such as anxiety, depression, and self-harm [47]. This suggests that when exploring the association between MHPs and offending among girls, it might be an advantage to define and examine different MHPs rather than disorders and diagnoses.

### Risk factors associated with both offending and MHPs

Different factors are often said to interact with each other to explain individuals’ crime involvement (e.g., [26, 49]). Three variables that previous research have identified as highly important for both youth MHPs and offending include the parent–child relationship, parental monitoring, and associations with peers (e.g., [24, 26, 49, 53]). Since these three variables are associated with both MHPs and offending, it can be hypothesised that they might affect the association between MHPs and offending. There is therefore a need to control for the effects of parent–child relationship, parental monitoring, and associations with peers when examining girls’ MHPs and offending.

### Parent–child relationship

Regarding the relationship between youth MHPs and parents, previous studies have found that a higher-quality relationship (with a positive, high-quality relationship between parents and youth being defined as characterised by support, warmth, attentive communication, behavioural consistency, and the absence of harsh punishment, rejection, and maltreatment (e.g., [43]) predicts higher self-esteem and lower depression [38], and that abusive and neglectful parenting is a predictor of adult mental illness (e.g., [53]). It has further been found that parental support was associated with lower levels of emotional and behavioural problems in adolescents [32] and that critical and unsupportive parenting was associated with an increased risk of depressive symptoms in adolescents [88]. It has further been suggested that girls have more expressive and communicative bonds (including attachment and monitoring) with parents than do boys [87], which might partly explain the gender gap in offending. Moreover, regarding relationships with offending, a study that, among other variables, examined gender, family, and offending indicated that low parental attachment is a much stronger predictor of violent crime among females than men [3].

### Parental monitoring

Also, as mentioned, parental monitoring has been found to affect both mental health and offending among youth; for example, it has been found that parental monitoring is associated with lower levels of youth behavioural problems and better mental health outcomes Dishion and McMahon [21], and that low parental monitoring is associated with increased delinquency and emotional problems in youth [7]. However, Hardie [33] found that the role of monitoring may decrease depending on the youth’s ability to exercise morality and self-control, which in turn can be affected by, for example, MHPs included in the externalising problem group [4]. There are thus clear indications that both the parent–child relationship and parental monitoring can affect both MHPs and offending; however, less is known from studies examining all variables together and whether they affect the association between MHPs and offending.

### Peers

Regarding youth peers and MHPs, it has been found that positive friendships are important; for example, they can reduce anxiety and depression [9], and that socialising with peers with disruptive and deviant behaviour carries a higher risk of individuals developing similar behaviours and adopting similar attitudes (e.g., [22, 49]) which can lead to increased emotional problems [22]. Also, Prinstein and Dodge [60] emphasise that peer influence plays a critical role in shaping adolescents' behaviours and mental health, and states that peer acceptance and the desire to fit in can lead to adopting harmful behaviours, contributing to mental health issues such as anxiety, depression, and substance abuse. Moreover, in relation to offending, it is widely known within the field of criminology that associations with antisocial and deviant peers are an important predictor of youth offending (e.g., [72, 82–89]). However, findings in studies vary across genders, for example, one study found deviant peers to be a better predictor among boys than girls who offend [58], but another study found that the effect of deviant peers on offending was similar among girls and boys [84], indicating the need for more research to understand the effect of deviant peers on offending among girls, and also to enable considering the effects in relation to MHPs and offending. As with parents, there are thus clear indications that associations with deviant peers can affect both MHPs and offending, but less is known from studies examining all variables together and whether deviant peers affect the association between MHPs and offending.

### Current study

From what has been presented above, we know that MHPs, to different degrees, seem to be associated with offending among girls and that this association may be influenced by factors such as parent–child relationship, parental monitoring, and peers they associate with. However, as previous research in this field is mainly based on clinical or juvenile justice samples, we need more knowledge on how offending and MHPs are associated among girls in a community-based sample. Addressing this in a community sample enhances the possibilities for early intervention by identifying problems before they escalate to a level where mental health care is needed or the young person becomes involved with the juvenile justice system.

The current study explores: (1) if some MHPs are more common among teen girls who reported that they have committed crime(s) compared to those who have not, (2) how different types of MHPs are associated with offending, and (3) whether these associations are affected when we control for parent–child relationship, parental monitoring, and peers.

## Method

### Sample

Data used in the study were drawn from the research project Malmö Individual and Neighbourhood Study (MINDS), which is modelled on the Peterborough Adolescent and Young Adult Development Study (e.g., Wikström et al., 2012) with some modifications to better meet the specific aims of the MINDS project and a Swedish context. The project was approved by the Swedish Regional Ethical Review Board in Lund (Dnr. 201/2007 and Dnr. 2014/802). It has a longitudinal design and follows a sample of 526 randomly selected adolescent boys and girls born in Malmö, Sweden, in 1995 (about 20% of the total cohort) and living there in 2007 (when the project was initiated). Three waves of data collection (excluding a pilot study with a smaller subsample) were completed when the adolescents were approximately 16, 17, and 19 years old. At ages 16 and 17, about 515 adolescents participated in the data collection; at age 19, the attrition rate increased, resulting in the number of participants dropping to 411. Data were collected using a self-report questionnaire and structured interviews, usually in small groups at the participants' schools. For a small number of cases, a postal survey was sent to those who could not be reached through the school (for a more detailed description of the project, see e.g., [18, 37]).

The current study included data on girls from the MINDS study who participated in the second and third wave of data collection when the participants were 16 and 17 (n = 240 girls) years old. Since relatively few girls reported that they had committed any crime (age 16, n = 59; age 17, n = 77), we merged the two waves to increase power, resulting in a total of 240 girls included in the analytical sample (i.e., 96% of the girls participating in wave two thus also participated in wave three, giving a small external dropout and missing values in the analytical sample). Wave one was excluded in the current study due to its pilot design, and wave four was excluded due to the increase in attrition and participants then being considerably older (19).

About 65% percent of the girls in the study lived with both their parents, which is in line with Swedish children on average [61]. Almost 40% of the girls have two foreign-born parents. This indicates an underrepresentation of participants with a foreign background, as the corresponding figure for the total cohort was about 50% [62]. There is also an overrepresentation of girls from the more affluent areas of Malmö.

Malmö is the third-largest city in Sweden, with approximately 360,000 inhabitants [45]. About one-third of the population in Malmö is born abroad, compared to 25% in the other two large cities and 20% in Sweden in total, and the population is relatively young, with about 20% being younger than 18 years. The percentage of inhabitants with higher education is above the national average; however, unemployment rates are also above the national average [45]. Like other large Swedish cities, Malmö has both affluent areas and disadvantaged neighbourhoods with lower socioeconomic status.

### Measures

The data employed in the current study were based on the self-report questionnaire.

### Dependent variable

Self-reported offending was measured with a self-report questionnaire with ten different crime items, including violence (e.g., assault and arson), property crime (e.g., burglary and vandalism), and drug crimes (if the participant had used any drugs). Committing property crimes (age 16 n = 50; age 17 n = 69) was more common across both waves of data collection than committing violent crimes (age 16 n = 9; age 17 n = 8) and using drugs (age 16 n = 8; age 17 n = 33) (see Appendix 1 for a detailed description of number of participants committing each crime type across the two different waves). Drug use almost exclusively involved the use of cannabis (wave two n = 8 and wave three n = 33), and due to the relativity small evidence of cannabis (compared to, for example, cocaine or amphetamine) (e.g., [20, 55]) increasing the risk of offending, we chose to not examine drug use as a risk factor of offending in the current sample and study, but only as a crime type. The crime types were added together into a variety scale by counting the crime types that each respondent had committed over the past 12 months. The variety scale was then dichotomised to represent whether a girl had committed any crime (= 1) or not (= 0). The crime scale was chosen to be dichotomised due to the skewed nature of the crime variable; a rather small number of girls had committed crimes, and in that way we addressed the problem of using a non-normally distributed variable in, for example, linear regressions (e.g. [27]).

### Independent variables

Youth MHPs were measured by using the Swedish version [71] of the self-report version of the Strength and Difficulties questionnaire (SDQ) [30]. SDQ is a widely used questionnaire, and the SDQ total difficulties sum has been found to be a psychometrically sound measure of overall child MHPs in studies from around the world [29]. However, the 25 items of SDQ can also be divided into five subscales with five different items each. The subscales have the intention of tapping into five different dimensions of mental health: emotional symptoms (alpha value for wave 2 = 0.68 and wave 3 = 0.67), conduct problems (alpha value for wave two = 0.50 and wave three = 0.45), hyperactivity (alpha value for wave two = 0.73 and wave three = 0.70), peer-related problems (alpha value for wave two = 0.45 and wave three = 0.48), and prosocial behaviour (alpha value for wave two = 0.63 and wave three = 0.56). SDQ can also be said to measure personality traits, instead of distinct MHPs; for example, traits of anxiety and depression. However, even though in the current study we are interested in problems and not traits, we argue the fit of the measure due to that the measured traits in turn give rise to MHPs associated with the traits. Prosocial behaviour stands out from the other four subscales in the way that it does not represent a problem but instead consists of variables that may have a positive effect on mental health [9]. Even though this subscale does not represent an MHP, it was chosen to be included in the analyses because it might give important information about whether prosocial behaviour decreases the risk of offending or affects the association between the other subscales and offending. Even though some of the subscales showed poor alpha-values (i.e., below 0.70), the five subscales were chosen to be used since they arguably have the possibility to tap into more distinct information about youth MHPS and have the potential to predict child mental disorders [29] and thus also to contribute with valuable information for the aim of the current study regarding examining if some MHPs are more common among girls who offend compared to those who do not, as well as to examine the association between different MHPs and offending. For both waves, there were a few missing values on SDQ items, and missing values were imputed with a subscale mean if the answers to no more than two of the items were missing in each subscale [63]. However, SDQ can also be divided into two broader subscales [29] of internalising problems (consisting of the two subscales of emotional symptoms and peer-related problems) (alpha value for wave two = 0.65 and wave three = 0.67), and externalising problems (consisting of the two subscales of conduct problems and hyperactivity/inattention) (alpha value for wave two = 0.74 and wave three = 0.71). The scales of internalising and externalising problems range from 0 to 20, and a high score indicates higher levels of MHPs. The broader subscales were also used in the current study, both because they have been suggested to better fit a low-risk community sample [29], as well as because much previous research has used these problem groups (thus opening the opportunity for comparisons with the results of the current study). For all subscales, a higher value indicated higher levels of MHPs (except for the subscale of prosocial behaviour, where it is the opposite).

### Control variables

To assess the potential effect of the control variables (parent–child relationship, parental monitoring, and deviant peers), three different measures were created. Two items were combined into a mean index of Parent–child relationship: ‘If you have a problem, or feel sad or disappointed, do you normally speak to any of your parents or stepparents?’ ranging from ‘yes, always’ (0) to ‘no, never’ (3), and ‘How often do you speak to your parents or step-parents about how you are doing in school and if you get along with your friends?’ ranging from ‘Every day/almost every day’ (0) to ‘never/almost never’ (3). Even though the scale preferably should have included a higher number of items, these were the only two items in the questionnaire that measured the parent–child relationship, while the other parent-related question fit better in the parental monitoring scale. There were overall a few missing values on the items. The parent–child relationship measure was scaled so that a higher mean value indicated a weaker relationship. The Cronbach’s alpha of the measure was 0.6 in both waves.

Parental monitoring refers to the adolescents’ own perception of to what extent their parents have knowledge about their whereabouts. Another way of describing parental monitoring is how and to what degree the parent can influence the adolescent’s view of different situations and what action alternatives they find possible (even if the parent is absent) [33]. In the current study, parental monitoring was measured using three different items: ‘When you are out on your own or with friends, do your parents or stepparents normally know what you are doing?’, ‘When you are out on your own or with friends, do your parents or stepparents normally know where you are?’, and ‘When you are out on your own or with friends, do your parents or stepparents normally know what friends you are with?’. The girls had to rate to what extent the parents had this information on a scale with four options, ranging from ‘no, never’ (3) to ‘yes, always’ (0). These items were combined into a mean index with a Cronbach’s alpha of 0.80 in wave two and 0.79 in wave three. There were overall a few missing values, and they were imputed with a subscale mean if the answers to no more than two of the items were missing. The parental monitoring was scaled so that a higher mean value indicated a lower degree of monitoring.

To measure association with deviant peers, six different items were combined into a mean index. Each item consisted of a statement regarding whether the adolescent’s closest friend or friends engaged in different sorts of antisocial behaviour (e.g., ‘Do any of your closest friend or friends shoplift or steal from other humans or stores?’ or ‘Do any of your closest friend or friends destroys things that do not belong to them; for example, break windows, scribble or beet the paint on cars?’ and the girls had to rate the frequency of the behaviour on a scale ranging from never (0) to very often/many times (3). There were overall a few missing values, and they were imputed with a subscale mean if the answers to no more than four of the items were missing. The deviant peers measure was scaled so that a higher mean value indicated a higher level of association with deviant peers. The Cronbach’s alpha of the measure was 0.78 in wave two and 0.76 in wave three.

All measures were created separately for each wave and then combined into one by taking the average, with the exception of offending, where scales were combined and dichotomised. A paired sample t-test showed no significant differences between the two waves in relation to internalising problems, externalising problems, or parent–child relationship. The level of parental monitoring increased from age 16 to age 17, just like association with deviant peers.

### Analytical strategy

First, a descriptive analysis, which presents mean values and standard deviations for all variables, was conducted. Second, to examine if some MHPs were more common among girls who reported that they had committed crime(s) compared to those who had not, bivariate analyses of differences in MHPs between girls who had committed any offence and those who had not committed any offence were conducted using the independent samples t-tests. Third, to explore associations between offending, MHPs, and control variables, as well as testing for multicollinearity, Pearson’s correlations were calculated between the study variables as well as testing VIF values. Lastly, a number of logistic regression models were estimated to address if associations between MHPs and offending were affected when controlling for parent–child relationship, parental monitoring, and peers. In the first model (Model 1), the five subscales of SDQ were added in the same model to examine whether the association with offending changed when all scales were included in the same model, giving knowledge of their odds ratios and associations with offending when compared to each other. Next (Model 2), parent–child relationship, parental monitoring, and deviant peers were added to the analysis to examine whether these variables affect the odds ratios, and thus the associations between MHPs and offending (Model 3). In the fourth and final model, the five smaller SDQ subscales were replaced with the broader scales measuring externalising and internalising problems. The broader subscales were tested in their own model because they were computed of the smaller subscales, and there would be an overlap in measurement if all scales were added in the same model. Moreover, testing the broader subscales is in line with the previous recommendation that the broader subscales better fit low-risk community samples [29]. Testing them separately from the five smaller subscales of SDQ further gave the possibility to examine if they had better predictive value (higher odds ratios) than the five smaller subscales of SDQ in the current sample.

In addition, we conducted a number of sensitivity analyses using crime as a continuous/count variable. Both OLS and negative binomial regressions yielded results very similar to the ones found in the logistic regressions, indicating the same associations between MHPs, control variables (parent–child relationship, parental monitoring, and deviant peers) and offending, supporting the use of the dichotomous crime variable and presenting the result from the logistic regressions.

All analyses were conducted in IBM SPSS Statistics 29.

## Results

Table 1 presents the result from the independent samples t-tests, conducted to examine research question one: Were MHPs more common among girls who reported that they had committed crime(s) compared to those who had not? The analyses show that, except for peer problems, there was a significant difference in MHPs between girls who reported any offence and those who had not offended (p < 0.05). Girls who had offended reported higher levels of emotional symptoms, hyperactivity, and conduct problems, and lower levels of prosocial behaviour. Consequently, they scored higher on the two broader subscales of internalising and externalising problems. Among the five SDQ subscales representing different dimensions of MHPs, hyperactivity showed the biggest differences in mean values between offenders and non-offenders (5.09, SD = 2.05, compared to 3.48, SD = 1.20), indicating that hyperactivity is the most common MHP among offending girls in the current sample. Furthermore, the bivariate analyses show that girls who offended reported weaker relationship with their parents, lower levels of parental monitoring, and more association with deviant peers.

**Table 1:** Difference in mean scores between girls who had offended and girls who had not offended

	Total sample (n = 240)	Offended (n = 87)	No offending (n = 127)	t-value
Emotional symptoms (0–10)	3.83 (2.04)	4.35 (2.05)	3.50 (2.05)	− 2.814***
Hyperactivity (0–10)	4.03 (2.15)	5.09 (2.03)	3.48 (1.20)	− 5.620***
Conduct problems (0–10)	4.03 (1.16)	2.22 (1.11)	1.33 (1.08)	− 5.798***
Peer problems (0–10)	1.59 (1.27)	1.72 (1.16)	1.52 (1.50)	− 1.030
Prosocial behaviour (0–10)	8.46 (1.21)	8.22 (1.22)	8.61 (1.21)	2.326*
Internalising problems (0–20)	5.43 (2.73)	6.07 (2.99)	5.07 (2.57)	− 2.528*
Externalising problems (0–20)	5.70 (2.92)	7.28 (2.62)	4.82 (2.68)	− 6.703***
Parent–child relationship (0–3)	1.00 (0.68)	1.24 (0.70)	0.89 (0.63)	− 3.723***
Parental monitoring (0–3)	0.87 (0.53)	1.08 (0.50)	0.74 (0.55)	− 4.619***
Deviant peers (0–3)	0.54 (0.43)	0.75 (0.45)	0.40 (0.36)	− 6.115***

Standard deviations are reported within brackets

*p < 0.05, **p < 0.01, ***p < 0.001

Table 2 presents the result from the bivariate analyses using Pearson’s correlation to examine research question two: how different types of MHPs are associated with offending. The findings showed that externalising problems, hyperactivity, and conduct problems had the highest correlation with offending, while internalising problems, emotional symptoms, and peer problems showed lower correlations with offending. Prosocial behaviour was the only variable with a negative correlation with offending. When testing for multicollinearity, no multicollinearity was found, with all VIF values below 1.6, and thus below the recommended cut of of 5 [66]. The results presented in Table 2 indicate that especially problems that are included in the group of externalising problems have important associations with offending, while problems included in the group of internalising problems seem less important in relation to girls’ offending.

**Table 2:** Correlation tests between MHPs, control variables and offending. N = 240

	Offending	Hyperactivity	Conduct problems	Emotiona lsymptoms	Peer problems	Prosocial behaviour	Internal-asing problems	Externa-lasing problems	Parental monitoring	Parent–child relationship	Deviant peers
Offending	1										
Hyperactivity	0.361**	1									
Conduct problems	0.372**	0.502**	1								
Emotional Symoptoms	0.189**	0.238**	0.126	1							
Peer Problems	0.074	0.016	0.188**	0.321**	1						
Prosocial behaviour	− 0.0158**	− 0.224**	− 0.328**	0.107	− 0.136*	1					
Internalsing Problems	0.176**	0.185**	0.181**	0.897**	0.707**	0.016	1				
Externalasing Problems	0.417**	0.938**	0.770**	0.226**	0.087	− 0.296**	0.209**	1			
Parental Monitoring	0.307**	0.206**	0.279**	0.200**	0.045	− 0.245**	0.170**	0.263**	1		
Parent–child relationship	0.252**	0.235**	0.182**	0.215**	0.080	− 0.249**	0.198**	0.246**	0.510**	1	
Deviant Peers	0.400**	0.358**	0.412**	0.082	0.023	− 0.157*	0.072	0.429**	0.406**	0.212**	1

*p < 0.01, **p < 0.001

Table 3 presents the three models of logistic regressions, conducted to examine both research question two: How do different types of MHPs associate with offending? and three: Are these associations affected when we control for parent–child relationship, parental monitoring, and peers? In the first model, when SDQ subscales were entered simultaneously, it was found that conduct problems, hyperactivity, and emotional symptoms were positively associated with offending, with conduct problems (OR = 2.05, CI = 1.55–2.70) and hyperactivity (OR = 1.46, CI = 1.26–1.70) showing the strongest associations. After including parental monitoring, parent–child relationship, and association with deviant peers in the second model, the positive associations between hyperactivity and offending remained (OR = 1.22, CI = 1.02–1.47), as did the association between conduct problems and offending (OR = 1.37, CI = 0.99–1.91, indicating that girls with these types of problems are more likely to offend regardless of the quality of their relationship with parents, level of parental monitoring, or their association with deviant peers. Regarding emotional problems, the association with offending was no longer significant after controlling for parent–child relationship, level of parental monitoring, and association with deviant peers. In the third and final model, using the externalising and internalising scales instead of the subscales of SDQ, a positive association between externalising problems and offending was found (OR = 1.27, CI = 1.12–1.44), also after controlling for parent–child relationship, parental monitoring, and association with deviant peers, indicating that externalising problems have important associations with girls’ offending, regardless of the quality of their relationship with parents, level of parental monitoring, or their association with deviant peers. Internalising problems were found to have no significant association with offending when controlling for parent–child relationship, parental monitoring, and association with deviant peers. However, the confidence intervals and p-value suggest that this association nearly reached statistical significance (OR = 1.09, CI = 0.96–1.22, p = 0.06), and results highlight challenges, which could be attributed to the rather small sample, in drawing definitive conclusions about the relationship between internalising problems and offending, especially after accounting for factors such as parental relationship quality, parental monitoring, and associations with deviant peers. In this final model, association with deviant peers was also associated with offending, indicating that girls with deviant peers were more likely to have committed any offence.

**Table 3:** Logistic regression predicting offending

	Model 1	Model 2 (Nagelkerke R Square = 0.348)	Model 3 (Nagelkerke R Square = 0.346)
	OR (CI)	OR (CI)	OR (CI)
Emotional symptoms	1.21 (1.06–1.39)*	1.10 (0.92–1.32)	
Hyperactivity	1.46 (1.26–1.70)**	1.22 (1.02–1.47)*	
Conduct problems	2.05 (1.55–2.70)**	1.37 (0.99–1.91)*	
Peer problems	1.12 (0.91–1.38)	1.05 (0.82–1.38)	
Prosocial behaviour	0.79 (0.61–0.96)*	0.98 (0.73–1.30)	
Externalising problems			1.27 (1.12–1.44)***
Internalising problems			1.09 (0.96–1.22)
Parent–child relationship		1.33 (0.78–2.23)	1.33 (0.78–2.25)
Parental monitoring		1.44 (0.68–3.05)	1.49 (0.71–3.11)
Deviant peers		4.65 (1.73–12.53)*	4.76 (1.77–12.747)*

Odds ratios (OR) and 95% confidence intervals (CI) in brackets. N = 240

*p < 0.05, **p < 0.01, ***p < 0.001

## Discussion

MHPs among youth is an important public health issue that not only influences the everyday life of those affected but also can have more far-reaching consequences. This study examined if some MHPs were more common among girls that had offended compared to those that had not. Further, the association between MPHs and offending was investigated, as well as how these associations were affected by the relationship between the girl and her parents, the level of parental monitoring, and association with deviant peers. Firstly, in the current study, almost 35% of the girls reported that they had committed an offence during the past year. Compared to the prevalence reported in the National School Survey on Crime, where just over 50% of the Swedish girls reported any crime involvement during the past 12 months [75], this is considerably lower. However, crime involvement among girls has increased over time. Among girls who had offended, levels of MHPs were higher than among those who had not offended, with the most pronounced differences found in relation to the single subscale of hyperactivity and the bigger subscale of externalising problems. Results from the logistic regressions showed that problems that are included in the group of externalising problems have important associations with offending among teen girls, and also when controlling for other types of MHPs and parent–child relationship, parental monitoring, and deviant peers. These results are in line with previous research from both the juvenile justice setting (e.g., [19, 73]) and research based on community samples that have found both high frequencies of externalising problems among girls who offend, as well as highlighted their importance for explaining the development of offending (e.g., [51]). Combining the study result regarding externalising problems together with Moffitt et. al.’s. findings (e.g., [49, 50, 51]) that youth that commit a high number of crimes and continue doing so in their adulthood often have externalising problems, and the fact that girls’ offending trajectories seem to be similar to those in mixed gender studies [8, 68], gives important knowledge for prevention strategies. Based on the findings, it can be argued that it is of great importance to screen for externalising problems among girls at a young age as a way of decreasing the risk of a negative development in both MHPs and offending, especially among girls that risk developing long-lasting criminality with a high number of offenses.

Regarding problems included in the group of internalising problems, findings from the current study showed mixed results, which is in line with findings from previous research. The t-tests showed that girls who had offended reported significantly higher levels of emotional symptoms and internalising problems than non-offenders. However, in the logistic regressions, no significant association remained between internalising problems (neither subscales nor the combined scale) and offending when analysed together with externalising behaviour, and after controlling for parent–child relationship, parental monitoring, and deviant peers. However, it should be noted that the trait of internalising problems was almost significant (with a p value of 0.06), and that emotional problems were significantly associated with offending before controlling for parent–child relationship, parental monitoring, and deviant peers. So, even though no firm conclusions can be drawn from the results regarding internalising problems, they partially support previous research from both the juvenile justice setting (e.g., [14, 76]) and community-based studies (e.g., [24, 64]) that have found that problems included in the group of internalising problems have had positive associations with girls’ offending. The result of the emotional problem subscale further supports previous research that has reported high levels of, for example, depression among girls who offend (e.g., [73]). It could be considered whether the results are affected by the rather small sample size, the fact that internalising problems seems to be increasing among girls in general [12], and that the differences between offenders and non-offenders might be smaller in a low-risk community sample, than if girls in a community sample had been compared to high-risk girls in the juvenile justice system. Moreover, findings from Van der Molen et al. [80], Siponen et al. [67], and Martin [47] are interesting in relation to the findings of internalising problems in the current study, with Martin [47] suggesting that ADHD in girls might be misdiagnosed and coexist with other MHPs, Siponen et al. [67] noticing that especially a comorbidity of different MHPs impact offending, and Van der Molen et al. [80] stating that high-risk girls with disruptive behaviour also had higher risks of depression and self-harm. Their findings, combined with the findings from the current study, indicate that some internalising problems might be common among offending girls and have important associations with girls’ offending, even though the associations seem to be more complex than between externalising problems and offending. The results further thus indicate that more research is needed to fully understand the association between internalising problems and girls’ offending, especially when combined with other MHPs and in relation to other important risk factors of offending,

Regarding the control variables of parent–child relationship, parental monitoring, and associations with deviant peers on associations between MHPs and offending, results from the t-tests support previous findings (e.g., [72, 83]), showing that girls who had offended had a weaker relationship with parents, experienced less monitoring, and had a higher level of antisocial peers than those who had not offended. As mentioned, regarding their effect on associations between MHPs and offending, results from the logistic regressions showed that externalising MHPs remained significant after controlling for parent–child relationship, parental monitoring, and association with deviant peers, indicating that the associations between externalising MHPs and offending still remain when parental relationship, parental monitoring and deviant peers are adjusted for. It is also worth noticing that deviant peers were significantly associated with offending, indicating that deviant peers are also an important risk factor when MHPs are considered. More research is needed to fully understand the seemingly-complex relationships between externalising problems, deviant peers, and offending. The associations between internalising problems and offending did, however, seem to be more sensitive to the effects of the control variables. Findings regarding the effect of the control variables on the association between MHPs thus implicate that MHPs, and especially externalising problems, must be addressed when working with offending girls, and also among girls with multiple problems in different domains of their lives.

### Methodological considerations

There are several limitations in the current study that need to be addressed. Firstly, the sample was rather small, with a small overall number of girls and an even smaller group of girls who had offended. However, this was addressed by combining two waves of data, giving a higher amount of crime occasions than if we had used only one wave. That the MINDS study represents about 20% of the total cohort might increase the risk of skewedness from the full population. An indication of this is the underrepresentation of participants with foreign backgrounds and from more disadvantaged neighbourhoods. This needs to be considered when drawing conclusions from the study findings. However, the fact that the results are in line with previous research indicates that they are valid, even though it could imply that we overestimate or underestimate certain associations. Moreover, even if the sample was small, it has some important qualities and strengths; it is a community sample (in contrast to the more common juvenile detention samples), which could provide important results from a previously understudied population. Secondly, all data in the study were collected through self-report questionnaires, which always comes with the risk of both over- and under-reporting and internal dropout. However, dropouts were not an issue in the present study, given the low number of missing data and cases. Further, using self-reported data also gives the important chance and possibility of measuring unreported adolescent offending and MHPS that are still unknown by the psychiatric healthcare system, which thus might be missing in official records. Thirdly and lastly, even though SDQ has been suggested to be useful for screening for MHPs and is used widely [81], it has also been argued to not be optimised for community samples, and the alpha values for some of the five subscales were below the recommended threshold of 0.7. However, in the present study, we also used the broader externalising and internalising subscales as suggested for community samples [29], which showed better alpha-values. Furthermore, the SDQ subscale of conduct problems (a subscale that is also included in the externalising problems scale) includes some behaviours that can be considered as criminal behaviours (e.g., fighting). This implies a potential overlap of measurement with the offending variables. However, conduct problems are recognised as mental health problems, as they are found, for example, in the DSM-5 [5] in, for example, the diagnosis of conduct disorder, and it is therefore important to address conduct problems as MHPs when guiding intervention and preventive measures. In relation to internalising problems, since previous research and results from the current study have indicated that different types of internalising problems might be of more importance for girls’ offending than previously discussed, using a more distinguished measurement giving more detailed information of different internalising problems than SDQ, could have been beneficial for the study and both enhanced the clarity and applicability of the study findings. Therefore, it is recommended to, in the future, focus on the development of such types of instruments.

To guide future research, it is recommended that more focus is put on better understanding the associations between especially girls’ internalising problems and offending. Since results regarding internalising problems have been mixed in both previous research and in the current study, more research is needed to understand how these problems are associated with offending, particularly since they are increasing among girls [12]. Further, variables like past trauma also need to be examined in association to MHPs and girls’ offending, and also in regard to externalising problems. Past trauma has, in previous research, repeatedly been associated with girls’ MHPs [16, 59] and offending [40]. Investigating this association might expand the existing knowledge of the complex associations between girls’ MHPs, variables in the social environment, and offending.

## Conclusion

The current study corroborates findings from previous research showing that MHPs have important correlations to offending among youth girls, even when studying a community sample rather than juvenile justice or clinical samples. Particularly, externalising problems were common among girls who had offended, associations that remained after controlling for parent–child relationship, parental monitoring, and associations with deviant peers, indicating that externalising problems are especially important in relation to girls’ offending. Also, internalising problems were more common among girls who had offended in comparison to girl who had not. However, the association between internalising problems and offending was not as strong and conclusive, and appears to be affected by both the occurrence of externalising problems and factors such as deviant peers and family factors. This calls for further research to fully understand this association. Nevertheless, in general terms, the results indicate that it is important to recognise and address MHPs among girls as part of crime prevention. Doing so may foster better conditions for effectively addressing both girls’ MHPs and offending, ultimately contributiong to improved lives for them.

## Supplementary Information

Supplementary Material 1.

## Data Availability

The data that supports the findings of this study are not publicly available due to privacy, ethical, and legal restrictions. The data can be available upon request to the corresponding author with the guarantee that privacy, ethical, and legal restrictions are maintained.

## References

[1] Abram KM, Teplin LA, McClelland GM, Dulcan MK. Comorbid psychiatric disorders in youth in juvenile detention. Arch Gen Psychiatry. 2003;60(11):1097–108. 10.1001/archpsyc.60.11.1097. 14609885 10.1001/archpsyc.60.11.1097 PMC2893728

[2] Ahmad SI, Hinshaw SP. Attention-deficit/hyperactivity disorder, trait impulsivity, and externalizing behavior in a longitudinal sample. J Abnorm Child Psychol. 2017;45(6):1077–89. 10.1007/s10802-016-0226-9. 27838891 10.1007/s10802-016-0226-9 PMC5429206

[3] Alarid L, Burton V, Cullen F. Gender and crime among felony offenders: assessing the generality of social control and differential association theories. J Res Crime Delinq. 2000;37:171–99. 10.1177/0022427800037002002.

[4] American Psychiatric Association. Diagnostic and statistical manual of mental disorders. 2013; 5th edn.

[5] American Psychiatric Association. What is mental illness? https://www.psychiatry.org/patients-families/what-is-mental-illness#:~:text=Mental%20illnesses%20are%20health%20conditions,nothing%20to%20be%20ashamed%20of. Retrieved at 2024-0903. 2024.

[6] Anderson DM, Cesur R, Tekin E. Youth depression and future criminal behaviour. Econ Inq. 2015;53:294–317. 10.3386/w18656.

[7] Andersson F. The female offender: patterning of antisocial and criminal behaviour over the life-course. Malmö högskola, Fakulteten för hälsa och samhälle, Institutionen för kriminologi: Malmö. 2013.

[8] Andersson F, Levander S, Svensson R, Torstensson Levander M. Sex differences in offending trajectories in a Swedish cohort. Crim Behav Ment Health. 2012;22:108–21. 10.1002/cbm.1822. 22311379 10.1002/cbm.1822

[9] Barrett PM, Farrell LJ, Ollendick TH, Dadds M. Long-term outcomes of an Australian universal prevention trial of anxiety and depression symptoms in children and youth: an evaluation of the friends program. J Clin Child Adolesc Psychol. 2006;35(3):403–11. 10.1207/s15374424jccp3503_5. 16836477 10.1207/s15374424jccp3503_5

[10] Basto-Pereira M, Farrington DP. Developmental predictors of offending and persistence in crime: a systematic review of meta-analyses. Aggress Violent Beh. 2022;65:1–11. 10.1016/j.avb.2022.101761.

[11] Beaudry G, Yu R, Långström N, Fazel S. An updated systematic review and meta-regression analysis: mental disorders among adolescents in juvenile detention and correctional facilities. J Am Acad Child Adolesc Psychiatry. 2021;60(1):46–60. 10.1016/j.jaac.2020.01.015. 32035113 10.1016/j.jaac.2020.01.015 PMC8222965

[12] Bor W, Dean AJ, Najman J, Hayatbakhsh R. Are child and adolescent mental health problems increasing in the 21st century? A systematic review. Aust N Z J Psychiatry. 2014;48(7):606–16. 10.1177/0004867414533834. 24829198 10.1177/0004867414533834

[13] Borschmann R, Janca E, Carter A, Willoughby M, Hughes N, Snow K, Stockings E, Hill NTM, Hocking J, Love A, Patton GC, Sawyer SM, Fazel S, Puljević C, Robinson J, Kinner SA. The health of adolescents in detention: a global scoping review. Lancet Public Health. 2020;2020(5):e114–26. 10.1016/s2468-2667(19)30217-8. 10.1016/S2468-2667(19)30217-8 PMC7025881 31954434

[14] Cauffman EA. A statewide screening of mental health symptoms among juvenile offenders in detention. J Am Acad Child Adolesc Psychiatry. 2004;43(4):430–9. 10.1097/00004583-200404000-00009. 15187803 10.1097/00004583-200404000-00009

[15] Cauffman E, Lexcen FJ, Goldweber A, Shulman EP, Grisso T. Gender differences in mental health symptoms among delinquent and community youth. Youth Viol Juvenile Just. 2007;5(3):287–307. 10.1177/1541204007301292.

[16] Chamberlain P, Moore KJ. Chaos and trauma in the lives of adolescent females with antisocial behavior and delinquency. J Aggression Maltreatment Trauma. 2008;6(1):79–108. 10.1300/j146v06n01_05.

[17] Chesney-Lind M. Trends in Girls’ Delinquency in the United States. In: Banwel S, Black L, Cecil DK, Djamba YK, Kimuna SR, Milne E, Seal L, Tenkorang EY, editors. The Emerald International handbook of feminist perspectives on women’s acts of violence. Leeds: Emerald Publishing Limited; 2023. p. 481–99. 10.1108/978-1-80382-255-620231032.

[18] Chrysoulakis AP. Situational sources of rule-breaking acts: an analytic criminology approach (PhD dissertation, Malmö universitet). 2022; 10.24834/isbn.9789178772797.

[19] Crick NR, Zahn-Waxler C. The development of psychopathology in females and males: current progress and future challenges. Dev Psychopathol. 2003;15:719–42. 10.1017/s095457940300035x. 14582938

[20] Degenhardt L, Hall W, Lynskey M. The relationship between cannabis use and other substance use in the Australian population. Addiction. 2003;98(11):1697–707. 10.1111/j.1360-0443.2003.00435.x.

[21] Dishion TJ, McMahon RJ. Parental monitoring and the prevention of child and adolescent problem behavior: a conceptual and empirical formulation. Clin Child Fam Psychol Rev. 1998;1(1):61–75. 10.1023/A:1021800432380. 11324078 10.1023/a:1021800432380

[22] Dishion TJ, Tipsord JM. Peer contagion in child and adolescent social and emotional development. Ann Rev Psychol. 2011;62:189–214. 19575606 10.1146/annurev.psych.093008.100412 PMC3523739

[23] Epinosa EM, Sorensen JR, Lopez MA. Youth pathways to placement: the influence of gender, mental health need and trauma on confinement in the juvenile justice system. J Youth Adolesc. 2013;42:1824–36. 10.1007/s10964-013-9981-x. 23824982 10.1007/s10964-013-9981-x

[24] Farrington DP. Development and life-course criminology: key theroetical and empirical issues—the 2002 Sutherland award address. Criminology. 2003;41:221–5. 10.1111/j.1745-9125.2003.tb00987.x.

[25] Farrington DP, et al. Key results from the Cambridge study in delinquent development. In: Farrington DP, et al., editors. The Cambridge handbook of criminology. Cambridge: Cambridge University Press; 2003.

[26] Farrington DP. Integrated developmental and life-course theories of offending. In: Farrington DP, editor. Advances in criminological theory. New York: Routledge; 2005. 10.4324/9780203788431-1.

[27] Field A. Discovering statistics using IBM SPSS statistics. 4th ed. New York: SAGE Publications; 2013.

[28] Goldstein NE, Arnold DH, Weil J, Mesiarik C, Peuschold D, Grisso T, et al. Comorbid symptom patterns in female juvenile offenders. Int J Law Psychiatry. 2003;26:565–82. 10.1016/s0160-2527(03)00087-6. 14522226 10.1016/S0160-2527(03)00087-6

[29] Goodman A, Lamping DL, Ploubidis GB. When to use broader internalising and externalising subscales instead of the hypothesised five subscales on the strengths and difficulties questionnaire (SDQ): data from British Parents, Teachers and Children. J Abnorm Child Psychol. 2010;38:1179–91. 10.1007/s10802-010-9434-x. 20623175 10.1007/s10802-010-9434-x

[30] Goodman R, Meltzer H, Bailey V. The Strengths and Difficulties Questionnaire: a pilot study on the validity of the self-report version. Eur Child Adolesc Psychiatry. 1998;7:125–30. 10.1080/0954026021000046137. 9826298 10.1007/s007870050057

[31] Grisso T, Barnum R, Fletcher KE, Cauffman E, Peuschold D. Massachusetts youth screening instrument for mental health needs of juvenile justice youths. J Am Acad Child Adolesc Psychiatry. 2001;40:541–8. 10.1097/00004583-200105000-00013. 11349698 10.1097/00004583-200105000-00013

[32] Guilherme A, et al. Parental support, psychological control, and behavioral control: associations with adolescent anxiety. J Fam Psychol. 2016;30(4):478–88. 10.1037/fam0000191.

[33] Hardie B. Why monitoring doesn’t always matter: The situational role of parental monitoring in adolescent crime. 2017. 10.17863/CAM.15484.

[34] Hidayati NO, Suryani S, Rahayuwati L, Dewi NS. Scoping review of mental health problems among female prisoners. Open Access Maced J Med Sci. 2021;9(6):80–4. 10.3889/oamjms.2021.7322.

[35] Hipwell AE, Loeber R, Stouthamer-Loeber M, Keenan K, White HR, Kroneman L. Characteristics of girls with early onset disruptive and antisocial behaviour. Crim Behav Ment Health. 2002;12:99–118. 10.1002/cbm.489. 12357260 10.1002/cbm.489

[36] Holmes J. Female offending; has there been an increase. NWS Bureau of Crime Statistics and Research. Australia. 2010.

[37] Ivert A-K. Adolescent mental health and utilisation of psychiatric care: the role of parental country of birth and neighbourhood of residence (PhD dissertation, Malmö University, Faculty of Health and Society). 2013.

[38] Johnson MD, Galambos NL. Paths to intimate relationship quality from parent-adolescent relations and mental health. Fam Relat. 2014;76:145–60. 10.1111/jomf.12074.

[39] Jung H, Herrenkohl TI, Lee JO, Hemphill SA, Heerde JA, Skinner ML. Gendered pathways from child abuse to adult crime through internalizing and externalizing behaviors in childhood and adolescence. J Interpers Violence. 2017;32(18):2724–50. 10.1177/0886260515596146. 26264725 10.1177/0886260515596146 PMC4991959

[40] Kerig PK, Becker SP. Trauma and girls’ delinquency. In: Miller S, Leve L, Kerig P, editors. Delinquent girls. New York: Springer; 2012.

[41] Lau TWI, Lim CG, Acharryya S, Lim-Ashworth N, Tan YR, Fung SSD. Gender differences in externalizing and internalizing problems in Singaporean children and adolescents with attention-deficit/hyperactivity disorder. Child Adolesc Psychiatry Ment Health. 2021;15(1):3. 10.21203/rs.3.rs-47463/v3. 33482840 10.1186/s13034-021-00356-8 PMC7825195

[42] Loeber R, Burke JD. Developmental pathways in juvenile externalizing and internalizing problems. J Child Psychol Psychiatry. 2011;52(4):441–76. 10.1111/j.1532-7795.2010.00713.x. 10.1111/j.1532-7795.2010.00713.x PMC3314340 22468115

[43] Loeber R, Farrington DP. From juvenile delinquency to adult crime: criminal careers. Justice Policy Prevent. 2012. 10.1093/acprof:oso/9780199828166.001.0001.

[44] Källmen H, Israelsson M, Wennberg P, Berman AH. Criminal behavior and mental health problems among adolescents: a cross-sectional study and description of prevention policy in Sweden. Crim Justice Ethics. 2023;42(2):158–77. 10.1080/0731129x.2023.2241765.

[45] Malmö Stad. Fakta och statistik/Befolkning. https://malmo.se/Fakta-och-statistik/Befolkning.html. 2024. Retrieved 2024-11-18.

[46] Mallett CA. Youthful offending and delinquency: the comorbid impact of maltreatment, mental health problems, and learning disabilities. Child Adolesc Soc Work J. 2014;31:369–92. 10.1007/s10560-013-0323-3.

[47] Martin J. Why are females less likely to be diagnosed with ADHD in childhood than males? Lancet Psychiatry. 2024;11(4):303–10. 10.1016/s2215-0366(24)00010-5. 38340761 10.1016/S2215-0366(24)00010-5

[48] McCabe KM, Hough RL. Youth with psychiatric disorders in juvenile detention: a national study. Psychiatr Serv. 2003;54(6):887–93. 10.1176/appi.ps.54.6.887.

[49] Moffitt TE. Adolecence-limited and life-course persistent antisocial behavior: a developmental taxonomy. Psychol Rev. 1993;100(4):674–701. 10.1037/0033-295x.100.4.674. 8255953

[50] Moffitt TE. Life-course-persistent versus adolescence-limited antisocial behavior. In: Cicchetti D, Cohen DJ, editors. Developmental psychopathology: risk, disorder, and adaptation. Wiley; 2006. p. 570–98.

[51] Moffitt TE, Caspi A, Rutter M, Silva PA. Sex differences in antisocial behavior: conduct disorder, delinquency and violence in the dunedin longitudinal study. Cambridge: Cambridge University Press; 2001.

[52] Mohr-Jensen C, Müller Bisgaard C, Kjæsgaard Boldsen S, Stienhausen C. Attention-deficit/hyperactivity disorder in childhood and adolescennce and the risk of crime in young adulthood in a Danish Nationwide Study. J Am Acad Child Adolesc Psychiatry. 2019;58(4):443–52. 10.1016/j.jaac.2018.11.016. 30768385 10.1016/j.jaac.2018.11.016

[53] Morgan Z, Brugha T, Fryers T, et al. The effects of parent–child relationships on later life mental health status in two national birth cohorts. Soc Psychiatry Psychiatr Epidemiol. 2012;47:1707–15. 10.1007/s00127-012-0481-1. 22327406 10.1007/s00127-012-0481-1

[54] Mulvey EP, Iselin AM. Pathways to desistance: a longitudinal study of serious adolescent offenders. U.S. Department of Justice, Office of Juvenile Justice and Delinquency Prevention. 2009.

[55] Murray RP, Lopez AD. Cannabis use and crime: does the evidence support the hypothesis? The Lancet. 2004;364(9449):1897–906. 10.1016/S0140-6736(04)17574-0.

[56] Myers WC, Burket RC, Lyles WB, Kemph JP. DSM-III diagnoses and offenses in commit-ted female juvenile delinquents. Bull Am Acad Psychiatry Law. 1990;18:47–54. 2328333

[57] Office of Juvenile Justice and Delinquency Prevention. Trends in youth arrests for violent crimes. Juvenile Justice Statistics: National Report Series Fact Sheet. August 2022. U.S Department of Justice, Office Justice Programs. 2022

[58] Piquero NL, Gover AR, MacDonald JM, Piquero AR. The influence of delinquen peers on delinquency: does gender matter? Youth Soc. 2005;36(3):251–75. 10.1177/0044118X04265652.

[59] Podgurski I, Lyons JS, Kisiel C, Griffin JG. Understanding bad girls: the role of trauma in antisocial behavior among female youth, residential treatment for children & youth. Resident Treat Child Youth. 2014;31(1):80–8. 10.1080/0886571X.2014.880275.

[60] Prinstein MJ, Dodge KA. Understanding peer influence in children and adolescents. Guilford Press; 2008.

[61] SCB. Barns boende 2023. Barns levnadsförhållanden 2025:2. 2023.

[62] SCB. Befolkning. https://www.scb.se/hitta-statistik/temaomraden/integration/befolkning/. Retrieved 2025-03-28. 2025.

[63] Sdqinfo. SDQ: Information for researchers and professionals about the Strength & Difficulties Questionnaires. Web. 2023. 10.1111/j.1475-3588.2007.00443_2.x 32811128

[64] Schwartz JA, Ziegler DA, Aisenberg D. Depression and delinquency in the Add Health study. J Youth Adolesc. 2006;35(5):649–57. 10.1007/s10964-006-9060-9.

[65] Selinus EN. Childhood signs of ADHD and psychosocial outcomes in adolescence—a longitudinal study of boys and girls. Stockholm: Karolinska institutet. 2015.

[66] Shrestha N. Detecting multicollinearity in regression analysis. Am J Appl Math Stat. 2020;8(2):39–42.

[67] Siponen R, Andersson A, Oskarsson S, Ångström A-K, Beckley AL, Fazel S, Tuvblad C. Psychiatric diagnoses and criminal convictions in youth: a population-based study of comorbidities of diagnoses. J Crim Justice. 2023. 10.1016/j.jcrimjus.2023.102114. 10.1016/j.jcrimjus.2022.102009 PMC7618064 40904604

[68] Sivertsson F, Carlsson C, Almquist YB, Brännström L. Offending trajectories from childhood to retirement age: findings from the Stockholm birth cohort study. J Crim Justice. 2024;91: 102155. 10.1016/j.jcrimjus.2024.102155.

[69] Snyder HN, Sickmund M. The Girls Study Group: Understanding Female Delinquency. U.S. Department of Justice, Office of Juvenile Justice and Delinquency Prevention. 2006.

[70] Stattin H, Kerr M. Parental monitoring: a reinterpretation. Child Dev. 2000;71(4):1072–85. 10.1111/1467-8624.00210. 11016567 10.1111/1467-8624.00210

[71] Svedin CG, Priebe G. The Strengths and Difficulties Questionnaire as a screening instrument in a community sample of high school seniors in Sweden. Nord J Psychiatry. 2008;62(3):225–32. 10.1080/08039480801984032. 18609029 10.1080/08039480801984032

[72] Svensson R, Oberwittler D. It’s not the time they spend, it’s what they do. The interaction between delinquent friends and unstructured routine activity on delinquency: findings from two countries. J Crim Just. 2010;38:1006–14. 10.1016/j.jcrimjus.2010.07.002.

[73] Teplin L, Abram K, McClelland G, Dulcan M, Mericle A. Psychiatric disorders in youth in juvenile detention. Arch Gen Psychiatry. 2002;59:1133–43. 10.1001/archpsyc.59.12.1133. 12470130 10.1001/archpsyc.59.12.1133 PMC2861992

[74] The Region of Skåne. Folkhälsorapport: Barn och unga i Skåne. Region Skåne, Enheten för samhällsanalys. 2021.

[75] The Swedish National Council for Crime Prevention. Skolundersökningen om brott: Om utsatthet för och delaktighet i brott. Rapport 2024:10. Stockholm. 2024.

[76] Trulson CR, Marquart JW, Mullings JL, Caeti TJ. In between adolescence and adulthood. Youth Viol Juvenile Justice. 2005;3:355–87. 10.1177/1541204005278802.

[77] Tye CS, Mullen PE. Mental disorders in female prisoners. Aust N Z J Psychiatry. 2006;40(3):266–71. 10.1080/j.1440-1614.2006.01784.x. 16476155 10.1080/j.1440-1614.2006.01784.x

[78] Tyler N, Myles H, Karadag B, Rogers G. An updated picture of the mental health needs of male and female prisoners in the UK: prevalence, comorbidity and gender differences. Soc Psychiatry Psychiatr Epidemiol. 2019;54(9):1143–52. 10.1007/s00127-019-01690-1. 30903239 10.1007/s00127-019-01690-1

[79] Underwood LA, Washington A. Mental Illness and Juvenile Offenders. Int J Environ Res Public Health. 2016;13(2):228. 10.1002/9781118524275.ejdj0146. 26901213 10.3390/ijerph13020228 PMC4772248

[80] Veysey B. Adolescent girls with mental health disorders involved with the juvenile justice system. USA: National Center for Mental Health and Juvenile Justice, Policy Research Associates; 2003.

[81] Vugteveen J, de Bildt A, Theunissen M, Reijneveld SA, Timmerman M. Validity aspects of the strengths and difficulties questionnaire (SDQ) adolescent self-report and parent-report versions among Dutch adolescents. Assessment. 2021;28(2):601–16. 10.1177/1073191119858416. 31257902 10.1177/1073191119858416 PMC7883005

[82] Warr M. Companions in crime: the social aspects of criminal conduct. Cambridge: Cambridge University Press; 2002.

[83] Weerman FM. Delinquent peers in context: a longitudinal network analysis of selection and influence effects. Criminology. 2011;49:253–86. 10.1111/j.1745-9125.2010.00223.x.

[84] Weerman FM, Hoeve M. Peers and delinquency among girls and boys: are sex differences in delinquency explained by peer factors? Eur J Criminol. 2012;9:228–44. 10.1177/1477370811435736.

[85] Wiens K, Bhattarai A, Pedram P, et al. A growing need for youth mental health services in Canada: examining trends in youth mental health from 2011 to 2018. Epidemiol Psychiatr Sci. 2020;29: e115. 10.1017/S2045796020000281. 32299531 10.1017/S2045796020000281 PMC7214527

[86] World Health Organization. Mental health. https://www.who.int/news-room/fact-sheets/detail/mental-health-strengthening-our-response<. Retrieved at 2024-09-03. 2022.

[87] Worthen MGF. Gender differences in parent–child bonding: implications for understanding the gender gap in delinquency. J Crime Justice. 2011;34:3–23. 10.1080/0735648x.2011.554744.

[88] Yap MBH, Pilkington PD, Ryan SM, Jorm AF. Parental factors associated with depression and anxiety in young people: a systematic review and meta-analysis. J Affect Disord. 2014;156:8–23. 10.1016/j.jad.2013.11.007. 24308895 10.1016/j.jad.2013.11.007

