# Supplementary material for: Girls, mental health problems, and offending: findings from a community sample
Source: Child Adolesc Psychiatry Ment Health. 2025 May 19;19:59. doi: 10.1186/s13034-025-00907-3 (PMC12090681; doi:10.1186/s13034-025-00907-3)
Supplement: Supplementary file 1 — Supplementary Material 1. [file 13034_2025_907_MOESM1_ESM.docx]

| Crime type | Wave two (total n=250) | Wave three (total n= 240) | Analytical sample (total n=240) |
| --- | --- | --- | --- |
| Theft from person | 22 | 23 | 31 |
| Theft from store | 33 | 24 | 41 |
| Vandalism | 16 | 29 | 31 |
| Arson | 6 | 27 | 28 |
| Robbery | 0 | 1 | 0 |
| Physical abuse | 9 | 8 | 13 |
| Burglary (residential) | 0 | 1 | 0 |
| Burglary (public building) | 0 | 2 | 2 |
| Car theft/burglary in car | 0 | 0 | 0 |
| Used drug(s) | 8 | 33 | 34 |
| Any crime | 59 | 77 | 87 |

**Appendix 1. Number of girls who reported committing each of the crime types in the crime scale used for the of the study’s analyses.**
